# Experiences of Patients Living in a Unique Leprosy Hospice in Greece: An Interpretative Phenomenological Analysis

**DOI:** 10.7759/cureus.66358

**Published:** 2024-08-07

**Authors:** Maria Nikoloudi, Evaggelia Bogdani, Ioanna Tsatsou, Alexandra Mantoudi, Kyriaki Mystakidou

**Affiliations:** 1 Pain Relief and Palliative Care Unit, Radiology Department, School of Medicine, Aretaieion University Hospital, National and Kapodistrian University of Athens, Athens, GRC; 2 People with Special Needs Department, Centre of Social Welfare of Attika, Athens, GRC; 3 One Day Clinic, Hellenic Airforce General Hospital, Athens, GRC; 4 College of Nursing, University of West Attica, Athens, GRC

**Keywords:** psychological impact of confinement, personal experiences, interpretative phenomenological analysis, chronic illness, leprosy

## Abstract

Background: Hansen's disease, or leprosy, has a long-standing presence in human history, and our study uniquely delves into the experiences of individuals who are among the last survivors of this condition in Greece. During the early 1930s, patients with Hansen's disease from Spinalonga, an isolated location in Crete, were moved to a medical facility in Athens. This event represents a significant historical change in the management and treatment of the disease. Following Spinalonga's closure, a Sanatorium emerged, evolving into Greece's sole Hansen's disease center and the present-day refuge for patients, underscoring the enduring stigma and abandonment associated with the disease.

Method: Our study, conducted through six interviews with unstructured schedules, provides a unique opportunity for these individuals to share personal insights, offering a profound understanding of their interpretations and experiences.

Results: Through interpretative phenomenological analysis, we unearthed four superordinate themes: the pivotal nature of the diagnosis, the visible impact of the disease on the body, the stigma associated with leprosy and its effects on individuals, and the significance of 'home' as a place of solace and acceptance.

Conclusions: These themes collectively depict the deep emotional trauma experienced by the participants, shedding light on the enduring impact of historical stressors, confinement practices, and the challenges of living with a devalued identity, shaping their profound sense of self.

## Introduction

Hansen's disease, also known as leprosy, is the contemporary medical term for a disease that has persisted throughout human history. It was named after the Norwegian physician G.H.A. Hansen, who identified the bacterium, Mycobacterium leprae, causing leprosy in 1873 [1]. Hansen's disease is a chronic infectious disease that affects the skin, peripheral nerves, mucosa of the upper respiratory tract, and the eyes [2]. If left untreated, it can cause progressive and permanent damage to the skin, nerves, limbs, and eyes.

The contemporary understanding of leprosy is viewed through a medical paradigm that separates it from its long history and the measures taken to protect healthy populations. Leprosy, which has been recognized since ancient times in civilizations such as China, Egypt, and India [1], has been characterized as "the epitome of stigmatization" against those affected by the disease [3]. Throughout history, individuals with leprosy were massively and unconditionally excluded from society, initially expelled from cities, and later confined to leprosaria outside inhabited areas. The dominant strategy was to exclude leprosy sufferers from societies and social life. Not only were they expelled from the social fabric, but they were also stripped of their possessions and forced to rely mainly on charity.

In Western Europe, the church managed leprosy due to its authority over life, health, and salvation, seeing it as a punishment for sin but also a path to salvation [4]. Orthodox Christianity took a more natural approach, focusing on earthly causes such as environment and lifestyle.

Between 1930 and 1935, patients with Hansen's disease from Spinalonga, an island in Crete, transitioned from a historic leper colony in the early-to-mid 20th century to the Infectious Diseases Hospital in Athens, now accommodating 34 patients. Entering the hospital, one will have to walk a long way until reaching the ward where the few witnesses to the disease are. In a landscape that looks more like a Soviet-style housing complex than an ordinary hospital, the signs hanging on the buildings confirm that something different is here. "Hansenian Association" writes one of them. This site symbolizes persistent stigma and abandonment. In Greece, leprosy was managed through isolation until multidrug therapy (MTD) emerged in 1952, shifting from exclusion to inclusion with the establishment of the Anti-leprosy Station [5]. Foucault noted this change [6], highlighting how society moved from isolating lepers to integrating them through constant supervision within various social structures.

Currently, in Greece, while there is a historical backdrop of research on leprosy's societal implications, there is a burgeoning interest in contemporary literature focusing on the cultural, social, and personal aspects of the disease within the Greek context. The literature situates leprosy within broader sociological frameworks, aiming to comprehend its implications for individuals and society at large, paving the way for a more comprehensive understanding of the lived experiences of those affected by this historically stigmatized condition. Our study contributes uniquely to this growing body of research as our participants are among the last survivors of the leprosy hospice in Greece, adding a distinctive dimension to the exploration of leprosy's impact on individuals and society in the contemporary Greek context.

Following the implementation of MDT, substantial advancements have been made in diminishing the prevalence of leprosy and curtailing new cases. Global approaches have developed in tandem with advancements aimed at lessening the burden of this disease [7]. Inspired by the decline in the number of cases undergoing treatment, the World Health Assembly adopted a resolution urging member states to expedite their endeavors toward the worldwide eradication of leprosy as a public health concern by 2000. While most countries achieved this milestone by 2010, strategies from 2006 to 2015 were concentrated on upholding superior leprosy services and prompt diagnosis [8]. The reduction in disease burden was assessed by observing grade-2 disability (G2D) or noticeable deformities in newly reported cases. From 2016 onward, strategies have incorporated efforts to diminish the stigmatization faced by individuals affected by leprosy.

The majority of publications on leprosy predominantly center on the examination of stigma, often relying on anecdotal evidence, case studies, and descriptive research. These investigations utilize a mix of qualitative and quantitative methods, incorporating tools such as questionnaires and newer scales to measure the impact of stigma on social participation, depression, and overall quality of life. However, a noticeable gap exists in the literature regarding intervention studies.

In addressing these research gaps, intersectionality offers a valuable perspective by analyzing social inequalities that arise when multiple attributes intersect in a specific context. To illustrate, women of low socioeconomic status may encounter distinct forms of stigma compared to men of higher status within the same geographical area. This approach becomes pivotal in comprehending the stigma associated with leprosy, as it sheds light on how various stigmatized conditions intersect and affect individuals uniquely. Discrimination, influenced by factors such as race, ethnicity, caste, documentation status, age, class, or gender, may also manifest in a given area. Exploring these intersections enriches our understanding of the complex dynamics surrounding leprosy-related stigma and its differential impact on diverse individuals within a community [9].

The global leprosy strategy for the years 2021-2030 emphasizes the need to expedite efforts toward achieving the objective of eradicating leprosy entirely, encompassing zero disease, zero disability, and zero stigma and discrimination [10]. This strategy aligns with the broader framework outlined in the neglected tropical diseases (NTDs) roadmap for the period 2021-2030. Within the NTD road map, leprosy is identified as one of the diseases earmarked for elimination, focusing on interrupting its transmission [11]. Data regarding leprosy in 2022 were gathered from 182 countries out of a total of 221 countries and territories, spanning all six WHO regions. This signifies a substantial increase compared to the 143 countries that had reported data in 2021. In 2022, reporting was as follows: 42 countries in the African Region (AFR), 39 in the Region of the Americas (AMR), 21 in the Eastern Mediterranean Region (EMR), 39 in the European Region (EUR), 11 in the South-East Asia Region (SEAR), and 30 in the Western Pacific Region (WPR).

In 2022, the global tally reported 174,087 new leprosy cases, indicating a detection rate of 21.8 cases per million population. This showcased a 23.8% increase from the 2021 count of 140,594. New cases were identified across all six WHO regions. SEAR constituted the majority, accounting for 71.4% of new cases, followed by AFR (12.6%), AMR (12.3%), EMR (2.2%), WPR (1.4%), and EUR (<1%) [12].

Over the period of 2013-2022, there was a global decline of 19.3% in new cases, with a 6% decrease noted up to 2019. The emergence of the COVID-19 pandemic resulted in a nearly one-third reduction in new case identifications between 2019 and 2021. Outside this significant downturn, the decrease in cases has generally been gradual. Efforts were being made to enhance diagnostic tools for early detection of leprosy [12]. This includes the use of molecular techniques and innovative methods to identify the bacteria causing leprosy.

There were ongoing research efforts towards developing a more effective vaccine against leprosy. Although the Bacillus Calmette-Guérin (BCG) vaccine offers some protection, it is not fully effective against leprosy [13].

Initiatives were underway to ensure that MDT - the primary treatment for leprosy - remains accessible and available to all affected populations. Efforts focused on reducing drug resistance and improving treatment outcomes [14]. Addressing social stigma and discrimination associated with leprosy remains a priority. Programs aimed at reducing stigma and integrating affected individuals into society were being emphasized [15].

Improvement in data collection and reporting systems for better surveillance and tracking of leprosy cases worldwide. Strengthening healthcare systems in endemic regions to ensure proper reporting and management. There were movements to integrate leprosy programs with broader health initiatives to improve overall healthcare for affected individuals and communities. Research and advancements in leprosy continue to evolve, emphasizing early detection, effective treatment, and addressing social issues related to the disease [12].

A study proposal on leprosy today needs to blend medical sociology and disability studies, diverging from the traditional medical model. Medical sociology delves into the personal experiences of those affected by chronic illness, broadening our understanding beyond just medical descriptions [16]. Disability studies, however, focus on societal roles in shaping these experiences, highlighting oppressive environments as the root cause [17].

This study aimed to investigate the experiences of individuals with leprosy, considering both personal perspectives and the impact of oppressive conditions they faced during confinement. The primary objective of this research is to contribute to a deeper understanding of how these individuals made sense of their personal experiences. We invited them to express themselves and describe the trajectory of their lives in order to identify "patterns of meaning-making" [18] as they reflected on their experiences. Our hypothesis was that, by uncovering the meaning-making patterns in their narratives, we would gain insight into essential human qualities associated with living through the conditions these individuals endured. Specifically, we intended to explore how individuals who were diagnosed with leprosy in the previous century and were required to relocate to leprosaria (asylums) made sense of their experiences. By examining the way, they spoke about their lives and themselves, we believed that the stories they shared would reveal their attempts to make sense of their experiences and that these stories held inherent value.

## Materials and methods

Phenomenology

Phenomenology, originating from the Greek term "phenomenon," has recently illuminated overlooked facets of human experience and revolutionized the way we approach philosophical inquiries. Unlike studies predominantly focused on causality, our methodology delves deeper into individuals' experiences, aiming to comprehend the cultural, social, and personal hurdles confronted by those enduring chronic illness - especially how it shapes their identities and daily existence.

Employing interpretative phenomenological analysis (IPA), we conducted in-depth interviews to unravel recurring themes and meanings within their narratives. This approach facilitated a profound grasp of the emotional toll associated with coping with leprosy.

This research adopts a qualitative approach to explore the experiences and interpretations of individuals grappling with chronic illness. In line with Willig et al. [19], qualitative research emphasizes understanding the depth and nuances of experiences rather than solely establishing causal links, distinguishing itself from the typical quantitative research focus.

Participants

Our research engaged individuals who had leprosy and were among the last survivors of leprosy in Greece. We sought their personal stories and reflections about their current lives, aiming to facilitate their reintegration into society. Notably, these relocations were enforced without adequate consideration for their psychological well-being, making our project significant as our participants are among the last to have experienced such relocations.

In collecting data, we conducted six unstructured interviews with individuals diagnosed with leprosy, previously confined in leprosaria in Greece during the 1940s, all residing at the Anti-leprosy Station in Athens. To safeguard their privacy, we replace the original names. Demographic details are presented in Table 1.

**Table 1:** Samples' demographic characteristics

Name	Age	Education	Marital status
Participant 1	66	Elementary	Widow, 3 children and grandchildren, remarried-divorced
Participant 2	78	Elementary	Single
Participant 3	91	Elementary	Divorced, 1 daughter and 3 grandchildren
Participant 4	82	Elementary	Widow, 3 children and grandchildren
Participant 5	88	Elementary	Single
Participant 6	83	Elementary	Single

Data collection and analysis

Our research was approved by the Administration Centre of Social Welfare of Attika and the Aretaieion Hospital Ethics Committee (license number: 10236/18-11-2018) and undertaken in 2018. Adhering to ethical guidelines outlined in the British Psychological Society Code of Human Research, potential participants were assured of confidentiality and anonymity. The study aimed to raise academic awareness of their experiences and address their needs. The six physically and mentally capable individuals who volunteered for the study provided verbal consent due to physical or literacy-related concerns, and interviews were recorded.

Participants were informed about the potential distress and their right to halt participation, except during the report-writing phase. Interviews, lasting an hour, involved two researchers encouraging open responses, while a third supervised.

For data analysis, IPA was employed, which involves the following fundamental steps: (1) transcribing the interview material and conducting multiple readings of it; (2) making initial observations, including descriptive and linguistic aspects, while checking initial assumptions; (3) developing emergent thematic propositions that encompass both the participants' words and the researcher's interpretation; (4) creating superordinate themes by identifying connections among the themes; and (5) triangulating the analysis of the data, involving three researchers.

Initially, the analysis was conducted in Greek, and then relevant sections were translated into English.

The results are the outcome of the fifth step in the qualitative data analysis (triangulation). The three researchers independently analyzed manually the data, following the same methodology, which included the study of interviews, initial notes, descriptive comments, linguistic comments, comprehension notes, the emergence of thematic units, seeking connections between themes, and finally the creation of superordinate themes. The researchers extensively discussed the results and ultimately arrived at a conclusion with four superordinate themes.

## Results

The date of the diagnosis: a stop in time (superordinate Theme 1)

A fundamental characteristic observed in all the interviews was the participants' inclination to employ a chronologically linear narrative structure. They began by recounting the sequence of events surrounding their diagnosis and placed particular emphasis on the date of their mandatory confinement. Their narratives were filled with factual details, yet notably lacked references to their own emotional experiences.

Participant 2 described: “Well listen, my father got sick and my mother also. In 1945 my father got sick and went to the leprosarium… I was left with my mother. In 1949, after four years, my mother also got sick and left me to my grandmother and my grandfather [...] my mother went to the leprosarium in 1949 I was going to school…and I became 13 and a half…[and] in March of 1953 my illness appeared, I had developed a mole and I had also here in my hands' scars called ‘fimata’. I went to the Health Center at [name oftown] and they told me 'you have the illness of your mother and your father'" (Participant 2, 1.1-1.10).

For Participant 6: “I got the illness when I was 19 years old… and after six months of tests, they found that actually I had the disease. Then I came here [the Anti-leprosy Station] and stayed for two years […] it happened right after I had paid for my military services that I was diagnosed ... all bad things came up one after another” (Participant 6, 1.8-1.20), implying that, as he embarked on a journey towards an independent life, he had to confront this terrible misfortune.

While recounting the details of her diagnosis and confinement, Participant 4 unfolded a narrative of her life where the ages mentioned did not reveal their underlying coherence. We speculated that she was calculating the standard five-year period of the bacterium's incubation. “It was when I was twelve years old and I was an intern in the housekeeping school and all other children were active but I was always reserved […] when I became fourteen I got married […] when I became eighteen I got my first child […] on my twenties I came here, I had my two children and I was pregnant on the third, almost ready to give birth. Since then, everything started: I was sick” (Participant 4, 1.3-1.10).

Participant 5 said that it was “In 1958, I was 28 years old when I came here."

Participant 3, also, recalled: “It was in 1950 May 9 […] when the trees blossom, as they say […] they tested us for the bacterium […] I was 23 years old, two years old was my girl, and they were calling me every two to three days for medical tests […] I asked what was going on and I was told that the medical board had seen my tests and could not find for sure that I was suffering from it […] then I went to a school full of doctors in Athens […]” (Participant 3, 2.24-2.40). It was there that his obligatory confinement was agreed.

Lastly, Participant 1, who was a child of patients in confinement and a patient herself, said: “I’ve been here since I was five years old. They brought us here from Spinalonga” (Participant 1, 1.5).

Our participants, who were the last survivors in Greece to have experienced the medical paradigm of exclusion and confinement as regulatory mechanisms for the control of leprosy in the previous century, provided us with detailed accounts of their diagnoses. These details were remembered vividly, including exact dates, procedures, and the individuals involved. The experiences were recounted without any further cognitive or emotional processing. They were presented as "raw" psychological material, leading us to believe that we are dealing with a trauma that has been left unaddressed and is now being reenacted without being properly processed. We can speculate that not only was emotional processing not supported and actively avoided by the doctors, nurses, and public officials of that time, but the patients themselves were not allowed to experience or express their emotions. They were pressured to confront the societal emergency and prioritize public hygiene. Emotionally, the patients remained in a state of shock.

This point is further supported by the following evidence: “... people were killing themselves at those times. Young men were killing themselves, young women... they were giving them the drug as to get well and they were keeping it and they were taking it altogether at night when others were asleep” (Participant 4, 3.26-3.28).

The fact that some individuals resorted to considering death as a solution reveals the extent of the unexpressed and unmanageable emotional pain they experienced because of receiving such an unfortunate diagnosis and being forced to live under new conditions.

Participant 3 contributed also: “... and I want to say that the state destroys people’s lives as it makes mistakes; people have killed themselves in here they were ill without a doubt, but they couldn’t bear it” (Participant 3, 3.18-3.20).

The marks of the illness on the body (superordinate Theme 2)

Living with an affected body due to leprosy was a common experience among the participants. However, the severity of symptoms resulting from the disease varied significantly among them.

Participant 5 said: “... the only symptom I had was my eyebrow which had fallen off […] and from that they suspected it, and also it had affected lightly my arm its nerves […] my entire left arm. I didn’t develop skin rash, I was always clean” (Participant 5, 2.27- 2.30).

Participant 5 revealed an important aspect of the manifestation of the disease: "Cleanliness" refers to the absence of visible marks that were betraying the disease.

Participant 1 and Participant 6 had no external signs of the disease, which allowed them to hide it even from their relatives:

“Those days, despite the battle inside me, there were no visible scars or marks to show it. Every day, I had learned to hide the truth well. I put on a normal face to fit in” (Participant 6, 2.23-2.26).

“... because I don’t have scars or else and I look normal, I have not experienced the scorn and avoidance of others, whilst others who are deformed [...] you see that others would run away from them [...] because it was happening to me when I was going somewhere with my mother, I could see that, and that was hurting me” (Participant 1, 4.18-4.23).

Participant’s 1 experiences with her parents, who had severe visible symptoms of the disease, were distressing. She described in detail:

“... my father and my mother were in a very bad condition, very very bad, very ugly condition, their noses had fallen off, their hands were like this [curved she demonstrated], their eyes were standing out. They were in a very ugly condition… there was no treatment then…[and] they were in very bad condition” (Participant 1, 1.30-1.34).

Participant 2 exhibited severe symptoms that manifested in his body, leading to the amputation of his leg. His description of the experience focused primarily on factual details, with minimal reference to the emotional processing he had undergone. It is possible to speculate that, as Participant 2 was the child of parents who also had severe visible symptoms, he was accustomed to living without a leg, as his father had lost his many years prior. However, the limited emotional processing we observed should not be dismissed, and further reflection on this matter is provided in the discussion.

“I had a corn in my foot and had gotten a bit hard, my aunt back in 1958 they were holding festivals with musical instruments, so my aunt came and invited me, I went to the Managing Board [of the leprosarium] and they gave me one week leave […] we went there we sat, we ate, we drunk I was young, I was 19 years old, I danced. She was my father’s sister and she started crying when she saw me dancing, and when the festivities were over the next day I saw a black blister ‘-oh my God what has come to me now’, I asked. Be sure since then [I had problems with my leg] since I was 19” (Participant 2, 3.1-3.11).

He continued narrating: “... my foot was opening and closing […] in 1988 we got our discharge with my mother and I had my leg, and we lived together [in their own house] for 10 years […] but then it got infected, it got swollen, by 1996 I couldn’t bear it any longer […] a cousin of mine got me to the airport in a wheelchair and I came here, the doctors saw me and they said we must cut it” (Participant 2, 3.29-3.41).

“... and I said to the doctor [just before he operated on his leg] ‘-as much further down as you can Doctor so that I can use an artificial leg […] cut it right down the knee’ and he listened to me, blessed may he be, and he cut it under the knee and he was visiting me every day to check on how I was doing […] and after a week he told me that ‘your leg is doing well’ […] ‘... thank you, doctor, very much’ I said. He had listened to me because if they had cut it above the knee I would have been useless. Now I put on my [artificial] leg and I walk very well” (Participant 2, 4.6-4.18).

The type of adaptation to their illness that our participants have achieved requires further commentary. Despite experiencing aversion and avoidance from healthy individuals, participants exhibited a common attitude towards their bodies characterized by tenderness and tranquillity, suggesting that they struggled with their illness rather than against it. We came to understand that, due to the imposed confinement and the avoidance by the healthy population, the patients formed communities that self-managed their needs. They built churches, established schools for their children, shops, and cultivated land, utilizing the physical capacities of each individual within the leprosarium. They made the best use of their bodies according to their individual abilities.

When Participant 2 proudly described his parents, who were also patients, he spoke of their abilities (his father had built a coffee store at the leprosarium, while his mother milked the goats), despite the deformities in their limbs. While describing this, Participant 2 used a grammatical expression - which does not have a direct equivalent in English - that conveys tenderness by adding the diminutive ending "-aki," which literally means something small. For example, his father's hands were referred to as "cherakia" (meaning little hands) (Participant 2, 1.35), and his amputated leg as "podaraki" (Participant 2, 2.15). These poignant expressions led us to realize that, for these individuals, the symptoms of the disease were not perceived as ugly or repulsive, but rather viewed with affection and even love.

Stigma: the "mark" of others on them (superordinate Theme 3)

The way others perceived them was of significant concern to our participants. We titled this overarching theme "the mark of others" based on the following words:

“I had gone bride to that village […] they heard I had the disease, and you understand the label was big… even prostitutes nowadays are in fashion, those days we were treated as if we had committed a crime, as if we had killed someone, to that extent others avoided us” (Participant 4, 1.19-1.23).

Participant 4 described a situation of extreme stigmatization faced by those with leprosy. They were not only avoided due to the fear of contracting the disease themselves, but the patients were also deemed morally responsible for having the illness. The patients' sense of moral responsibility stemmed from the level of avoidance and repulsion they experienced from others' reactions. Participant 6 invented a happy self to cope: “The laughter and stories I shared were a cover, a way to keep my secret safe” (Participant 6, 2.27).

Participant 1, when discussing her parents who were significantly impacted by visible symptoms of the disease, further substantiates this point: “Repulsion is hard; is hard to see the other’s repulsion towards you” (Participant 1, 4.25).

The psychological impact of being accepted or avoided by others was central to the accounts of all participants. On the other hand, the emphasis placed on the love and affection they received from others reflects the same psychological process, questioning the level of acceptance the patients experienced in their social interactions. Participant's 5 experience serves as confirmation of this point:

“... I have many people whom I know and come by every Sunday, and they sit here and drink coffee and all my cups come down from the shelves and I’m used to having people around me” (Participant 5, 1.14-1.16).

Details such as “all cups being used” by others could easily be discarded as unimportant. Participant 5, however, kept on returning to this issue, as in: “... and I was taking the big pot [...] I was making the ‘dolmadakia’ [a traditional Greek dish], I was cooking them and taking them with me, and I was going to their house -it was near here- and they were eating them all” (Participant 5, 4.17-4.18).

For Participant 5, all these behaviors exhibited by others held significant meaning, and we could speculate that what these behaviors meant to her was that she was not feared, avoided, or seen as repulsive by others. Her friends would use her cups, drink the coffee she offered them, or eat from her cooking, and through their actions, she reassured herself that she was no longer a threat to others. The paradigm from the past, where leprosy was considered a deadly menace, clashed with the contemporary paradigm of leprosy as a manageable disease. Participant 5 was enjoying the fearless company of others in her present, while Participant 4 still vividly remembered the unforgettable mistreatment she endured from others in the past.

“... my nerves broke down, out of crying ... this loneliness... only consolation God first, and then the telly; I was not going out from the house because they were criticising me. Since they were criticising me wherever I was going I stopped going out... for as long as my husband was alive, he was going out to do the shopping, I was not going out at all... I got claustrophobic” (Participant 4, 2.19-2.24).

The participants did not provide elaborate intellectual explanations regarding the stigma of leprosy. Instead, they shared their personal experiences of stigmatization, which had impacted them in different ways. They had devised individual solutions to cope with the stigma, ranging from hiding the truth as Participant 6 did; emotional detachment from others, as in Participant's 4 case; to an equally distorted attitude exhibited by Participant 5. Participant 5, driven by her desire for acceptance, seemed to overlook the true intentions of others. She often spoke about a loving family that invited her to spend time with them, but it became evident between the lines that Participant 5 was more or less serving them rather than being treated as an equal friend.

“... they didn’t have a dishwasher, then, and I was doing the dishes, and I was standing by the sink for hours” (Participant 5, 4.21).

Home is where you feel comfortable being (superordinate Theme 4)

Extracts from our participants' narratives about their experiences of living in the institution formed the fourth overarching theme of our analysis. A shared characteristic among all participants was that they viewed the institution as their home, or rather, as the place where they truly belonged.

Participant 5, in particular, expressed a strong sense of belonging that began right from the moment she was admitted to the leprosarium. She described her feelings as follows:

“In 1958, I was 28 years old when I came here. We had an excellent doctor, Markianos was his name, the best in the Balkans I heard saying … there was a room in the hospital and we went there we the new ones and there were twenty young doctors […] the doctor saw us […] and he said this girl can leave, “-do you want to leave” he asked me, “-no Sir, I don’t want to leave”; because it was then that Father Eumenios had come, a wonderful soul […] and I was going and helping [in the Church] […] I had gotten used to living here [. ] I don’t want to [leave] although my relatives love me but I’m used to living here” (Participant 5, 1.1-1.16).

Participant 2 also communicated belongingness:

Interviewer: "When you were diagnosed you were very young, 10 years old…"

Participant 2: "13 and a half"

Interviewer: "How did you feel about it?"

Participant 2: "... em I was young I didn’t care. I went home, to my mother and my father.”

The leprosarium of obligatory confinement, where Participant's 2 father and mother resided, was the place that Participant 2 considered his home, and it was where he truly belonged.

Moreover, the institution not only served as a place of belonging but also provided a sense of safety and refuge. In cases of misfortune encountered in the outside world after their "obligatory" release from the asylums, the institution became a fantasy haven for the patients, a shelter from the challenges of the outside world. Participant 4 expressed this sentiment in the following words: “...em years went by and one morning I woke up and I found my husband dead in bed, after his death everything vanished” (Participant 4, 2.27-2.28).

She tried living in a different country with her children but she could not cope:

“I didn’t like it because sadness was covering me, I was becoming very sad because it rained a lot and the humidity made my bones... it was crashing my bones, I couldn’t live there. I talked to my older son saying that I’ll leave to return to Athens where we have a house, and if I can’t live there I’ll go to a monastery...my children believed me because they knew I loved the Church... yet I came here” (Participant 4, 2.31-2.37).

Participant’s 1 misfortunes in her outside life, also, rushed up her return. She gave her own explanation:

“... in 1982 after my house got on fire, I returned here and I re-registered myself [...] I was thinking that it would be difficult to find another house and I was wondering whether the owners would allow me to have my icons, if they would allow my dolls ... whilst here you sort of don’t have to think about electricity, water, or rent ... since it might be that in a year’s time they ask you to empty the house and you start all over again” (Participant 1, 5.35-5.40).

In this theme, we highlighted the challenges faced by these individuals in different aspects of independent living. They struggled with accepting and adapting to loss, as well as managing tasks related to independent living, such as running a household. It is worth noting that all the patients had been granted pensions when the medical approach of exclusion was reassessed, and their release was organized. However, these individuals had developed a deep attachment to the institution, considering it their true home - a home where they belonged unconditionally, just as they were.

## Discussion

We invited participants who were the last to have experienced both medical paradigms toward leprosy exclusion, followed by inclusion, to reflect on their experiences. Our aim was to foster a conversation that encouraged their reflexivity while minimizing our intrusions. We believed that the interaction prompted by our guiding questions would unveil the issues most important to them. The research design produced intriguing findings, revealing insights derived from the words and concerns expressed by our participants.

Upon reflection of our findings, it becomes evident that these individuals were significantly traumatized, which made it challenging for them to distance themselves from the long history of events, dates, vivid memories, and the people involved. Notably, the presence of trauma hindered their ability to abstract their experiences beyond their factual nature. Through providing detailed accounts of their experiences, participants conveyed not just their life stories but also seemed to relive the described events as if they were unfolding in the present moment. This characteristic aligns with the psychoanalytic model of therapy, emphasizing its centrality in the emotional world of traumatized individuals. Memories of acutely stressful and traumatic events are typically highly detailed, consistently vivid, and relatively reliable [20]. Since the work of Breuer and Freud in 1895, traumatic memory has been conceptualized as a foreign entity within the psychic framework, overwhelming the ego and compelling its compulsive revisiting in an attempt to "bind the excitation and reinstate the pleasure principle" [20].

Our argument posits that these individuals, due to the deprivation of their individuality throughout the history of leprosy treatment, experienced significant trauma. They were exposed to an excessive number of stressors that overwhelmed their emotional resilience. This assertion is supported by numerous studies that confirm the high prevalence of comorbidity between leprosy and psychiatric disorders [21]. We observed that all participants, in one way or another, distorted their situations. For example, Participant 5 embellished her experiences to seek acceptance from others, thus hindering her ability to recognize that she was being taken advantage of. Participant 2, on the other hand, perceived the leprosarium as his "home," failing to acknowledge the trauma associated with his condition. Participant 3, in denial of his leprosy diagnosis, disconnected himself from the trauma, Participant 6 lived a life trying to hide the truth about the disease and his true feelings, while Participant 4, overwhelmed by sadness, struggled to lead an independent life outside the institution. Despite the improvement in their living conditions over the years, they remained emotionally overwhelmed.

These individuals had to adapt to a series of stressors, primarily their illness. They needed to reconcile their sick bodies and the physical losses they experienced with their familiar sense of self, establishing a new relationship between the two [22]. Life within the leprosaria played a constructive role in this process. These institutions functioned as organized communities with fundamental structures [23]. Each individual contributed to the communal life through their respective abilities, such as serving, cooking, washing, or providing care for others. Within these communities, the stigmatizing negative labels associated with leprosy lost their power.

Furthermore, these individuals had to adapt to the devalued identity imposed upon them by society due to leprosy. Upon diagnosis, societal practices assign devalued and degrading qualities to those affected by the disease. It is worth noting that individuals were not seen as suffering from leprosy or having leprosy; they were labeled as "lepers," highlighting the pervasive impact of this label on a person's identity. Hasselblad [24] emphasized the interplay between self-identity, how individuals perceive themselves, social identity, and how others perceive individuals with leprosy. Our findings revealed how participants grappled with the stigma associated with leprosy [15], seeking to decipher how others perceived them and whether they accepted or rejected them. Even during our interactions, we observed their vigilance in interpreting our gaze upon them.

Regarding the changes in the self of our participants, as they lived with a stigmatizing disease, our findings revealed a notable silence regarding self-references. Charmaz [22] emphasized the importance of attending to the silences in participants' accounts in research, and we would add that we should be mindful of what remains unspoken in their narratives. What our participants remained silent about was their own self-referential experiences and their struggles to reestablish a sense of self and social identity.

While reviewing the literature to inform our study, we came across qualitative research [25], which explored how individuals make sense of their chronic illness-related pain and its impact on their sense of self. In comparing our findings to theirs, we observed that our participants did not actively search for explanations for their condition, nor did they compare themselves to their previous selves or others. The exploration of self was largely silenced among our participants. As Gergen [26] highlighted, the self is constructed through language when individuals are granted the right to be heard. Gergen's definition implies two processes: the individual's desire to be heard and the provision of space for their voice. Our participants were denied the right to express themselves and to construct a sense of self through language. Under the weight of leprosy, their individualities were silenced, and they were not afforded the opportunity to voice themselves as independent selves. Consequently, they had to find alternative ways to construct their identities beyond language. Participant's 1 room, which she invited us to see, depicted a different space for self-construction. In that space where she could find and connect with herself, dolls and teddy bears filled the room - a manifestation of childhood desires for tenderness and tranquillity.

Our study aligns with prior research that emphasizes the broad impact of leprosy, extending beyond physical effects to encompass social, emotional, and economic challenges faced by affected individuals [27,28].

Limitations

The study included only six individuals relocated due to leprosy in the 1940s, all from a specific Athens facility. This small group might not fully represent the diverse experiences of all relocated individuals. It did not consider viewpoints from various locations or situations, possibly creating a one-sided view of relocations like those to Spinalonga. Additionally, relying on memories from many years ago might have altered or influenced participants' recollections due to changing historical contexts.

## Conclusions

Our study confirmed previous research findings, revealing that participants engaged with their illness through the cultural meaning system surrounding leprosy. They navigated societal meanings and experienced severe stigmatization, leading to social exclusion and the loss of their previous lives. The biographical disruption caused by leprosy led to the establishment of new routines and a devalued identity, profoundly affecting their social identities and overall sense of self.

By exploring the experiential worlds of the last survivors of leprosy in Greece, our research aimed to avoid reducing their experiences to mere "personal tragedies." We emphasized the crucial role of social aspects, such as meanings and practices, in framing the context of living with leprosy over the past two centuries. This comprehensive approach highlights the interplay between individual experiences and the broader social context, offering invaluable lessons for empathetic and holistic patient care.
